# Control of humanoid robot via motion-onset visual evoked potentials

**DOI:** 10.3389/fnsys.2014.00247

**Published:** 2015-01-09

**Authors:** Wei Li, Mengfan Li, Jing Zhao

**Affiliations:** ^1^Department of Computer and Electrical Engineering and Computer Science, California State UniversityBakersfield, CA, USA; ^2^School of Electrical Engineering and Automation, Tianjin UniversityTianjin, China

**Keywords:** brain robot interaction, mind-controlled humanoid robot, N200 potentials, humanoid robot behavior, visual feedback

## Abstract

This paper investigates controlling humanoid robot behavior via motion-onset specific N200 potentials. In this study, N200 potentials are induced by moving a blue bar through robot images intuitively representing robot behaviors to be controlled with mind. We present the individual impact of each subject on N200 potentials and discuss how to deal with individuality to obtain a high accuracy. The study results document the off-line average accuracy of 93% for hitting targets across over five subjects, so we use this major component of the motion-onset visual evoked potential (mVEP) to code people's mental activities and to perform two types of on-line operation tasks: navigating a humanoid robot in an office environment with an obstacle and picking-up an object. We discuss the factors that affect the on-line control success rate and the total time for completing an on-line operation task.

## Introduction

Event related potentials (ERPs) are able to set up a communication between external stimuli and people's cognitive tasks. Assigning specific meanings to visual stimuli allows to “read” people's mind by identifying a target stimulus related with their attention (Lebedev and Nicolelis, 2006). A P300 model based on visual attention mechanism (Jin et al., 2012) is commonly used in the ERP-based brain-computer-interfaces (BCIs). When we evaluated the P300 model on Cerebot—a mind-controlled humanoid robot (Li et al., 2011, 2012), we noted some issues of this model (Li et al., 2013a,b). First, this model needs to flash a visual stimulus by growing its visual contrast, which easily causes people's visual fatigue (Hong et al., 2009). Second, the P300 potential is correlated with both the people's attention allocation (Farwell and Donchin, 1988) and the biological determinants of cognitive operation (Polich and Kok, 1995), so the people's states and experimental environments significantly affect the P300 signal quality. Considering the problems above, we investigate a N200 potential-based robot brain interaction (BRI) model.

The stimulus appearing in moving a bar through images instead of flashing the images (Heinrich, 2007) induces a N200 potential with a negative deflection occurring at 180–325 ms post-stimulus (Patel and Azzam, 2005). The N200 potential is an involuntary component that less depends on people's attention, and even people's fixation can induce this kind of potential (Frensel and Neubert, 2010). N200 potentials may promise a useful BCI model for controlling external devices due to the interface's low requirements of luminance and contrast, the large amplitude of the induced brain signal, and the low individual difference of mVEP (Schaeff et al., 2012). A N200-speller based Internet browser (Liu et al., 2010) reports that the N200 stimulus interface causes less visual discomfort and the induced N200 potential is more stable and less affected by the adaption effect. A BCI system is presented by combining mVEP and P300 potentials (Jin et al., 2012).

Comparing with manipulators and mobile robots, humanoid robots are more advanced as they are created to imitate some of the same physical and mental tasks that humans undergo daily (Hirai et al., 1998), but control of humanoid robots is much more complex. Humanoid robots are being developed to perform a wide range of complex tasks like personal assistance, where they should be able to assist the sick and elderly, and dirty or dangerous jobs. Recently, controlling a humanoid robot via brainwaves becomes more attractive. Bell et al. (2008) and Choi et al. (Choi and Jo, 2013) used ERPs to select an object as a target that a humanoid robot should reach, while our study focuses on telepresence control of humanoid robot behavior via the N200 potentials, including walking in an environment with obstacles and picking-up an object. The challenge to develop an ERP-based model is to make a trade-off between improving the classification accuracy and shortening the intervals between commands in controlling the humanoid robot in real time under the limited information transfer rate (ITR) (Wolpaw et al., 2000).

When investigating the P300-based BRI models (Li et al., 2013a,b), we noticed that the N200 components in brainwaves acquired from our experiments were stable and their amplitudes were relative high. The prominent shape of ERP is very helpful to build feature vectors for improving the classification accuracy. In this article, we propose a BRI model based on the N200 potentials. In order to acquire N200 potentials with high quality, we design an interface by replacing characters in a regular speller with robot images representing robot behaviors to be controlled with mind. We evaluate this N200-based model by telepresence controlling a humanoid robot with live video feedback. We analyze the N200 potentials induced by the experiment procedure to suggest how to improve the proposed model.

The paper is organized as follows. In Section Materials and Methods, we present the materials and methods for this study, including our mind-controlled humanoid robot system-Cerebot, the detailed experiment procedure, and the method for analyzing and recognizing the N200 potentials. In section Results, we off-line analyze the N200 potentials elicited by the experiment procedure and present the model performance regarding the accuracy, the ITR and the practical bit rate (PBR) (Jin et al., 2012). In this section, we apply the N200-based model to telepresence control a humanoid robot to accomplish two types of tasks. In section Discussion, we discuss the factors that affect the performance of the on-line control operation tasks and draw some conclusions.

## Materials and methods

### Cerebot

Cerebot is a mind-controlled humanoid robot platform (Li et al., 2011, 2012). Cerebus™ is the neural signal acquisition system in this platform. It is able to record both invasive and noninvasive neural signals and its processor can deal with on-line signal pre-processing, such as filtering and line noise removing. The platform uses two kinds of humanoid robots. The first one is a NAO humanoid robot made by Aldebaran in France [http://www.aldebaran.com/en]. The other one is a KT-X PC humanoid robot made by Kumotek in USA [http://kumotek.com]. Both of the humanoid robots with high degree of freedoms (DOFs) are equipped with microphones, a camera, a sonar rangefinder, etc., to provide environment information. The Cerebot platform can be used: first, to challenge brainwave-based methods since control of a humanoid robot with full body movements is difficult; second, to evaluate different methods for controlling a humanoid robot under a uniform platform; third, to testify neuroscience assumptions; fourth, to investigate the effect of telepresence control on the subject's mental activities. In this study, we implement the N200 model on Cerebot to on-line navigate a NAO humanoid robot in an office environment and to pick-up an object based on live videos sent back by the camera embedded in the robot.

**Figure 1:**
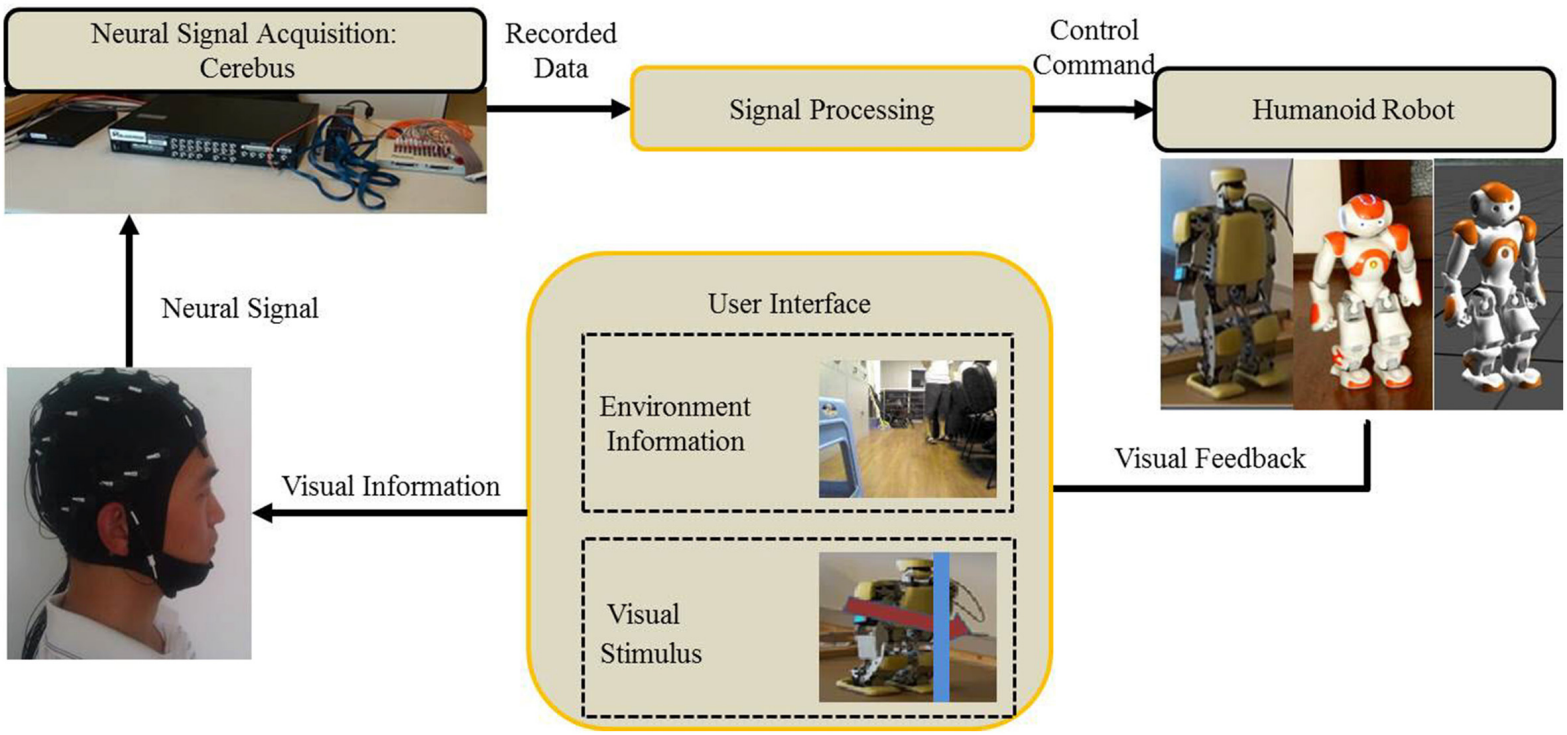
**A N200 model based mind-controlled humanoid robot**.

The control architecture of Cerebot is developed under the OpenViBE-based programming environment. OpenViBE is a free and open-source software platform for designing, testing, and using brain-computer interfaces. It consists of a set of modules devoted to the acquisition, pre-processing, processing, and visualization of cerebral data, as well as to the interaction with Virtual Reality (VR) (Renard et al., 2010). It offers a powerful interface named “Virtual-Reality Peripheral Network (VRPN)” to communicate with other scripts programmed in Matlab or Python. In the Cerebot system (Zhao et al., 2013), OpenViBE integrates the signal acquisition section, the signal processing section, and the control section, as shown in Figure 1. In order to control the NAO robot via N200 potentials, an OpenViBE module generates a serial of visual stimuli in a random order to a subject who focuses on a target stimulus (a robot image) that codes the subject's mental activity. Cerebus™ records brainwaves and sends them to the signal processing section to pre-process them, to extract their features of N200 potentials, and to classify them according to the codes of the subject mental activities. Once the subject mental activity is identified, its corresponding control command is sent to the control section to activate the robot behavior.

### N200 model

#### Flow diagram

**Figure 2:**
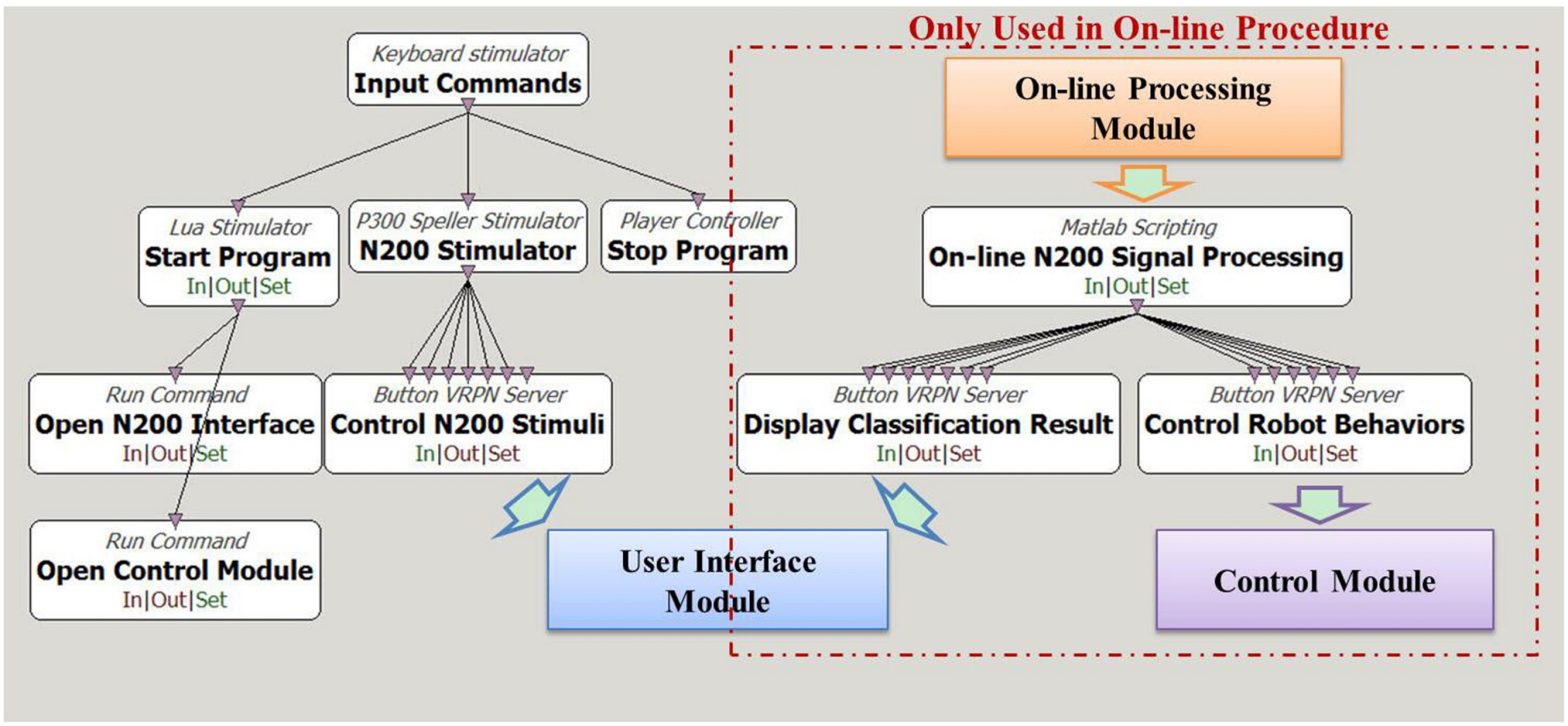
**Diagram of N200 model under the OpenViBE programming environment**. The white boxes are functions provided by the OpenViBE software package, and the colored boxes are the modules for the N200 model. The boxes enclosed in the red dashed rectangle are only for the on-line control procedure.

We implemented the N200 model on Cerebot under the OpenViBE programming environment. Figure 2 shows the model flow diagram. The white boxes in the diagram are the toolboxes provided by the OpenViBE packages, while the colored boxes are modules developed in C++ or Matlab. The arrows in the diagram indicate data flow paths. The N200 model uses the Start Program toolbox and the Open N200 Interface toolbox in Figure 2 to start up the User Interface module programmed in C++. To start an experiment, the N200 model uses the N200 Stimulator toolbox provided by OpenViBE to determine the settings, e.g., the stimulating timeline of the visual stimuli, for the User Interface module. The communication from the N200 Stimulator toolbox to the User Interface module is established via VRPN. The User Interface module activates six robot images in random order as the visual stimuli to evoke N200 potentials and sends their event markers to Cerebus™ via serial port to lock the time point at which the relevant image is activated.

The model is designed for both off-line analyzing the acquired N200 potentials and on-line controlling the humanoid robot via brainwaves. The off-line procedure uses the Central software to record the brain signals acquired from the Cerebus™ system. The off-line N200 signal processing scripts developed in Matlab, which are not displayed in Figure 2, process the recorded data to analyze the brain signal features and to investigate classification algorithms. In the on-line procedure, the On-line N200 Signal Processing toolbox starts up the On-line Processing module via the Matlab engine to load the configured parameters, to acquire the brain signals from Cerebus™ in real-time and to classify them according to the N200 feature vectors generated during the off-line process. The Display Classification Result toolbox displays the hit visual stimulus on the user interface by framing the corresponding robot image. The Control Robot Behavior toolbox converts the classification result to its corresponding command for Control Module, which activates the behavior to be controlled with mind. Figure 2 shows how the toolboxes and modules exchange data with each other under the OpenViBE environment.

#### Interface and protocol for acquiring N200 potentials

**Figure 3:**
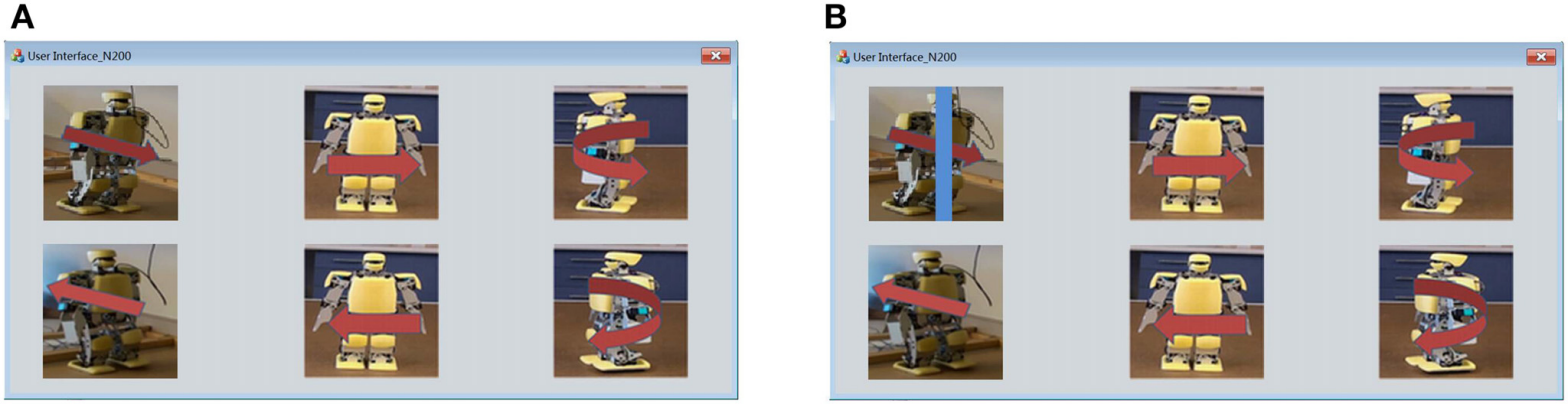
**(A)** User interface of visual stimuli is a 2 × 3 matrix of six robot images depicting six humanoid robot behaviors. **(B)** The visual stimulus for walking forward is activated by using a blue bar to scan the robot walking forward image from left to right.

Figure 3A shows the user interface with a 2 × 3 matrix of images. These images taken from the real represent six robot behaviors: walking forward, walking backward, shifting left, shifting right, turning left and turning right. We attached red arrows on the images to make the meanings of the images more impressive. In order to induce N200 potentials, a blue bar scans an image from right to left. Figure 3B shows that the blue bar is moving on the image to activate the visual stimulus of robot working forward.

**Figure 4:**
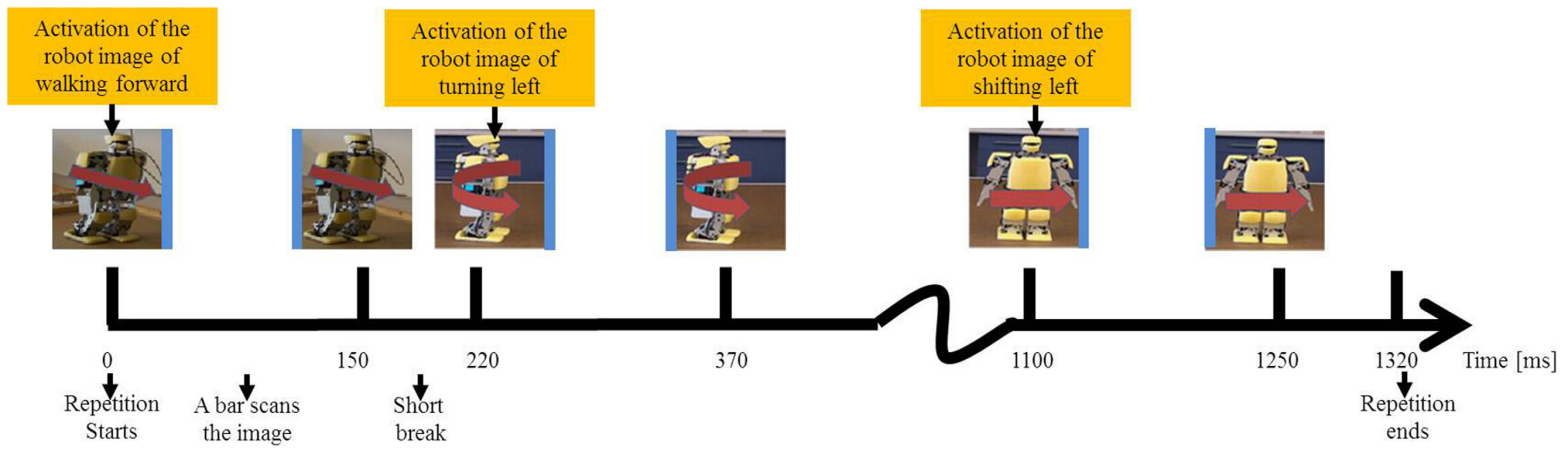
**Protocol of inducing N200 potentials activates the six visual stimuli one by one in a random order**.

We applied the Single Character (SC) method (Guger et al., 2009) to randomly activate the images one by one with the probability of 1/6. The duration of the stimulus onset asynchrony (SOA) (Wei and Luo, 2010) is 220 ms consisting of 150 ms for scanning and 70 ms for a break between two consecutive activations. A repetition is defined as a process in which each image is scanned. The repetition duration is 220 ms × 6 = 1320 ms. Figure 4 displays the entire process of a repetition. It uses the blue bar to scan the image of walking forward for 150 ms and takes a break for 70 ms, and afterward it uses the blue bar to scan the image of turning left, and so on. The repetition is completed after 1320 ms as the blue bar has scanned all the six images. Each image is randomly selected for scanning in a repetition, so the subject cannot predict which image will be the next visual stimulus. A number of repetitions constitute a trial in which the blue bar repeatedly scans each image for several times.

#### Experiment procedure

We conducted experiments in a quiet environment and asked subjects to sit in a comfortable armchair. The horizontal distance between the armchair and a monitor was 70 cm. The monitor was a 22-inch LCD one with a resolution of 1280× 1024 pixels. The electroencephalogram (EEG) signals were recorded at 1000 Hz from 32 surface channels using an EEG cap according to the “International 10-20 system.” The linked mastoids were reference channels and the channel AFz was the ground channel.

Five subjects (one female, four males, aged 26–28) signed a written informed consent to participate in experiments. Tianjin medical university general hospital ethics committee gives the oral approval of the consent form and experimental procedure before any subjects participated. All of their visual acuities were normal or corrected to normal. Three of them had no prior experience on the experiments. Each subject conducted 360 repetitions, i.e., 36 trials. In each trial, the subject selected a robot image as a target stimulus according to his/her mental activity. During a trial, the subject needed to focus on the target stimulus, tried his/her best to ignore the non-target stimuli, and had to avoid making any movements. Cerebus™ received the visual stimuli and simultaneously recorded the evoked potentials.

### Signal analysis and feature extraction

#### Signal analysis

We analyzed the recorded brain signals to extract their features. First, the brain signals were cut into epochs that were simultaneously with the visual stimuli. The length of each epoch was from post-stimulus 0 ms to post-stimulus 800 ms to cover the potentials. Because the delta (0.5–4 Hz) and theta (4–7.5 Hz) oscillation contribute to the N200 potentials (Karakas et al., 2000a) and the drift mainly appears in a low band, a digital band-pass filter with 1–10 Hz was chosen to process the epochs. Second, we removed the signal drift by the method of common average reference (CAR). Finally, the epochs were divided into two groups: the epochs induced by the target stimulus and the ones induced by the non-target stimulus, and then both the groups of the epochs were averaged, respectively.

#### Feature extraction and classification

Being able to represent the feature of brain signals in low dimension space can reduce the amount of computation (Bian and Zhang, 2000). According to Shannon's theorem, we were able to reduce the dimension of the feature vectors by down-sampling the data epochs from 1000 to 20 Hz (Krusienski et al., 2008). We used the N200 signal processing scripts to remove noises from the epoch from post-stimulus 100–500 ms and to average the epochs induced by the same visual stimulus in a trial. The brain signals from a single channel yielded an 8-dimension [(500-100)/1000 × 20] feature vector. If *n* channels were used to extract the features, the total dimension of a vector was 8 × *n*. The selected channel number depended on the characteristics of individual subject's brain signals.

We adopted the Fisher's linear discriminant analysis (FLDA) as a two-class classifier to discover which visual stimulus was the target one by checking each feature vector. The idea of this algorithm is to find the optimal direction of projections that groups the vectors with the same features into a class (Mika et al., 1999) as bellow:

$$\begin{array}{l} \;w=S_{w}{}^{-1}\left(M_{1}-M_{2}\right)\\ S_{w}=S_{1}+S_{2}\\ S_{i}=\sum_{x_{k}\in {\text{X}}_{i}}\left(x_{k}-M_{i}\right){\left(x_{k}-M_{i}\right)}^{T},i=1,2\\ M_{i}=\frac{1}{n_{i}}\sum_{x_{k}\in {\text{X}}_{i}}x_{k},i=1,2 \end{array}$$

where **x***_k_* is the feature vector, X*_i_* represents the class set and *n_i_* is the number of feature vectors in the *i*th class.

We trained the FLDA classifier using the feature vectors and tested it by the brain signals recorded in a trial that established six feature vectors according to the six visual stimuli. The trained classifier processed each feature vector successively and outputted the classified value. The classifier outputted no control command if it classified the six features vectors as non-targets or outputted a control command if it classified one or more feature vectors as targets.

## Results

### Induced N200 potentials

**Figure 5:**
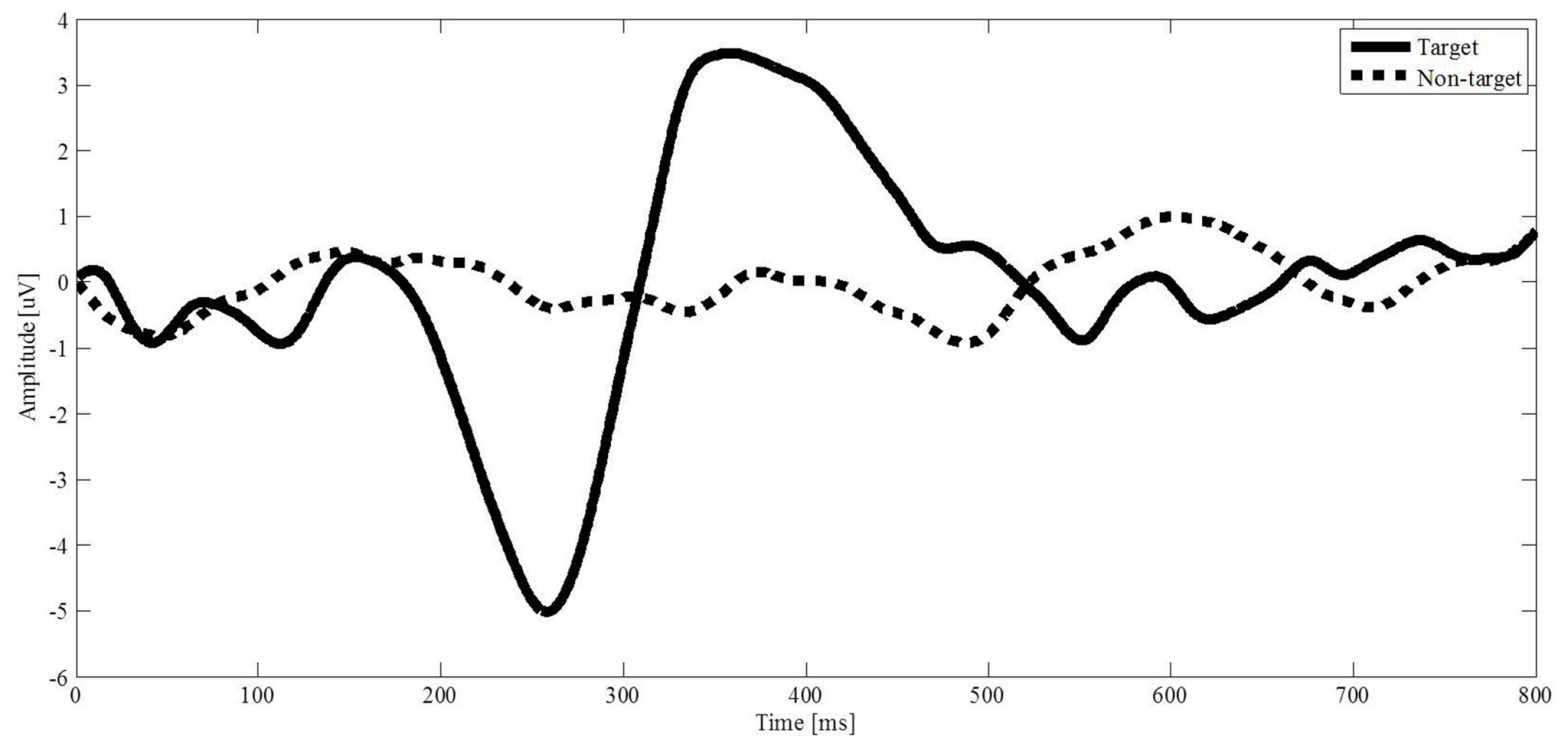
**Evoked potentials from channel P3 are averaged**. The solid curve is the brainwave of a target stimulus, and the dotted curve is the brainwave of a non-target stimulus.

The solid and dotted curves in Figure 5 represent the average brain signals induced by the target stimulus and non-target stimulus from channel P3, respectively. The brain signal induced by the target stimulus appears with a sharp negative deflection with amplitude of 5 uV at 258 ms and a positive deflection with amplitude of 3.5 uV at 358 ms. The negative deflection is known as the N200 potential that is the response to scanning over the target image by the blue bar. The positive deflection resembles the P300 potential. The brain signals induced by the non-target stimuli do not appear with obvious deflection. The results demonstrate that the designed interface can induce N200 potentials by scanning over a target image with the blue bar. The negative deflection provides a recognizable feature of the N200 potential for us to classify the brain signals.

**Figure 6:**
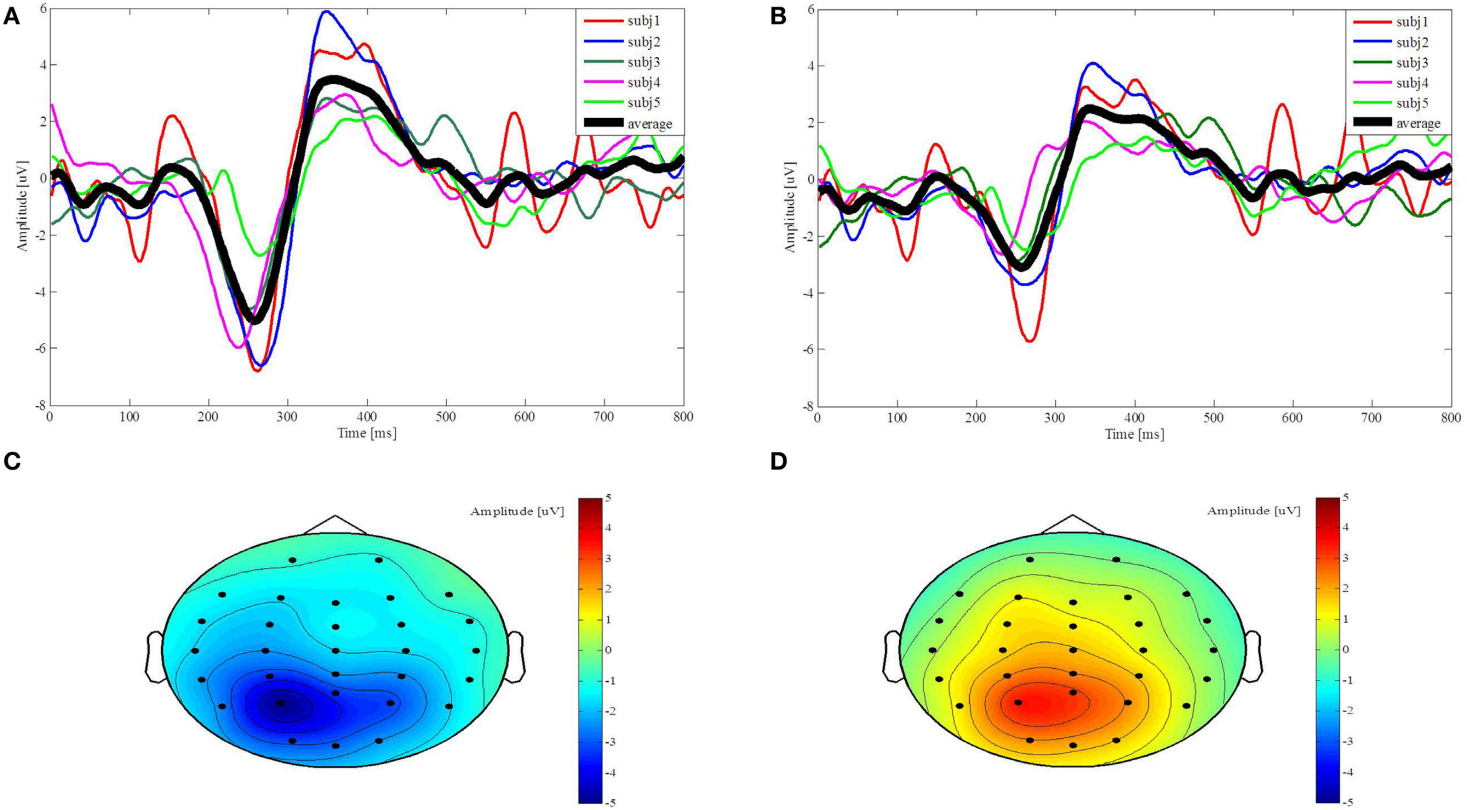
**Evoked potentials from channel P3 and CP3 and their topographies along with time points**. **(A,B)** The colorful thin curves represent the five subjects' N200 and P300 potentials from post-stimulus 0–800 ms, respectively, and the black curve represents the average potentials over the five subjects. **(C,D)** The amplitude topographic maps of the average brain signals across five subjects are plotted at 258 ms and 358 ms.

We plot the average brain signals acquired from each subject through channels P3 and CP3 to discuss their individual difference, as shown in Figures 6A,B. The black thick curve is the average N200 potentials across the five subjects, while the other thin color curves are the N200 potentials from the individual subject. The N200 potential amplitudes acquired from the subj5, represented by the green curves in Figures 6A,B, are smaller than the others. The latencies of the N200 potentials acquired by the subj4, represented by the pink curves in Figures 6A,B, is 20 ms shorter than the average latency. The N200 potentials induced from the subjects show different amplitude distributions over channels P3 and CP3. For example, the N200 potential amplitude acquired from the subj2 through channel P3 is larger than the one through channel CP3, represented by the blue curves in Figures 6A,B, while the N200 potentials amplitudes acquired from the subj5 through channels P3 and CP3 are very close. The amplitude topographic maps show the distribution of the induced potentials. Figures 6C,D represent the amplitudes of average brain signals at 258 ms and 358 ms after the visual stimuli, respectively. The darker the red color indicates the positive amplitude the greater, and the darker the blue color indicates the negative amplitude the greater. Figure 6C shows that the largest amplitude of N200 potentials mainly appears in the temporal-parietal area near channel P3, while Figure 6D shows that the P300 potentials mainly distribute in the parietal area. We draw two following remarks. First, although the N200 potentials acquired from the subjects are slightly different, they exhibit the N200 potentials' features that are important to control the robot behavior. Second, the N200 and P300 potentials mainly appear in the parietal and temporal areas in a window of post-stimulus 100–500 ms and the channels from which the recognizable N200 potentials are acquired vary due to the individuality, so we determine time windows and select the best channels for the individual subject for processing N200 potentials to control the humanoid robot.

The electrical potentials caused by eye movement and blinking can be much larger than the ERPs (Joyce et al., 2004) and propagate across the scalp, so these electrical potentials may distort the ERPs, but their impact on the ERPs decreases as a distance from the frontal increases. Because the induced N200 potentials mainly appear in the temporal—parietal area that is far from the frontal, the distortion of the N200 potentials induced by using a blue bar scanning visual stimulus images is irrelevant. In this study, therefore, we directly apply the induced N200 potentials to on-line control the humanoid robot.

However, the distortion of the P300 potentials induced by flashing stimulus images may be significant since the P300 potentials mainly appear in the parietal and central areas (Iturrate et al., 2009). Consequently, an additional algorithm has to be designed to remove eye artifacts to ensure high classification accuracy.

### Off-line evaluation

This subsection evaluates the off-line performance of the N200 model based on the classifier accuracy, which is the ratio between the trials detected correctly over the total trials, the ITR and the PBR.

**Figure 7:**
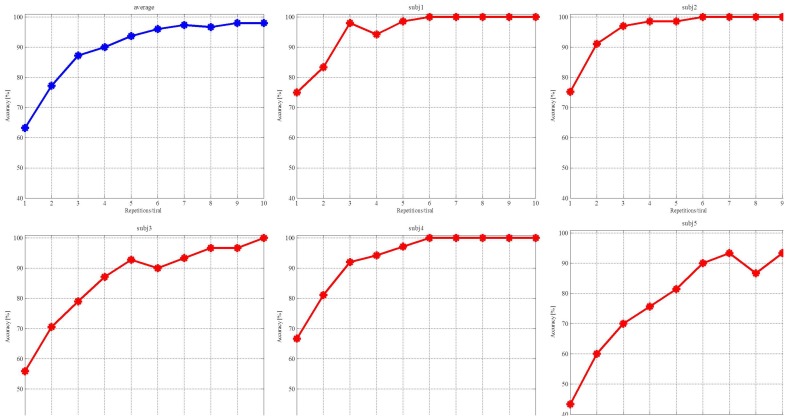
**Red curves are the accuracies achieved by the individual subjects and the blue curve is their average accuracy**.

We averaged the classified accuracy of each subject by the 10-fold cross-validation (Liu et al., 2010). The first step was to determine the candidate channels based on the subject's amplitude topographic map. The second step was to calculate the classified accuracy of each channel and their combinations. The third step was to select the combination that yielded the highest accuracy as the optimal channels for the individual subject, listed in Table 1. The three subjects denoted by **N** had no prior BCI experience on the experiments, while the other two subjects denoted by **Y** had prior BCI experience. Figure 7 depicts the average accuracy for each subject vs. the number of repetitions. The accuracy is clearly increased when increasing the number from 1 to 10. The accuracies of the four subjects reach 100%, and three of them reach 100% with 6 repetitions. The accuracy of subj5 is slightly low. ITR measures the information rate per minute by taking the accuracy and the time needed to classify a visual stimulus described as

$$B=\left[{\text{log}}_{2}N+{\text{log}}_{2}P+\left(1-P\right)\times {\text{log}}_{2}\left(\frac{1-P}{N-1}\right)\right]\times M$$

where *N* = 6 stands for 6 visual stimuli in the user interface, *P* is the classifier accuracy, *M* is the number of outputting commands in a minute (McFarland et al., 2003). The index, PBR, estimates the practical speed of a system, by considering each error classification that needs to be corrected by additional selections (Jin et al., 2012) as below:

PBR=B×[1−2×(1−P)]

**Table 1:** **The off-line performance**.

**Subject**	**Optimal channels**	**Accuracy %**	**ITR (bits/min)**	**PBR (bits/min)**
subj1 (Y)	P3, P4, Pz, CP3	98.6	22.22	21.58
subj2 (N)	P3, P4, Pz, CP3	98.6	22.22	21.58
subj3 (N)	P3, P7, CP3	92.9	18.61	15.95
subj4 (Y)	P3, P4, Pz,	97.1	21.19	19.98
subj5 (N)	P3, CPz	81.4	13.28	8.34
Mean	–	93.7	19.50	17.49

PBR is meaningful only when *P*≥50%. Table 1 lists the five subjects' accuracies, ITRs and PBRs. For evaluating the off-line ITR, we set both the interval between the repetitions and the one between the trials as 0 ms (Jin et al., 2011), so *M* is 9.09 (0.22 × 6 × 5 = 6.60 s, 60.00/6.60 ≈ 9.09) when the repetition number is 5. In Table 1, each subject's ITR is larger than 10 bits/min. ITR increases when the accuracy is increased. The highest accuracy of 98.57% yields the largest ITR of 22.22 bits/min. PBR is smaller than ITR due to [1 − 2 × (1 − *P*)] ≤ 1. The large accuracy reduces the difference between ITR and PBR. Clearly, a high accuracy allows the subject to correct the error quickly and to realize his/her intention accurately, while a low accuracy needs the subject to spend much time to correct an incorrect command. Improving the accuracy needs to increase the number of repetitions in a trial, i.e., to increase the control cycle, so a trade-off between the accuracy and control cycle speed must be considered according to a task requirement.

### Case studies

In order to validate the developed N200 model, the subjects controlled on-line the NAO humanoid robot to accomplish two popular tasks in robotics research: to navigate the NAO robot to walk with obstacle avoidance and to control the NAO robot to pick up an object. These tasks are challenging because the subjects need live video feedback from a camera embedded in the NAO robot to activate appropriate robot behavior.

**Figure 8:**
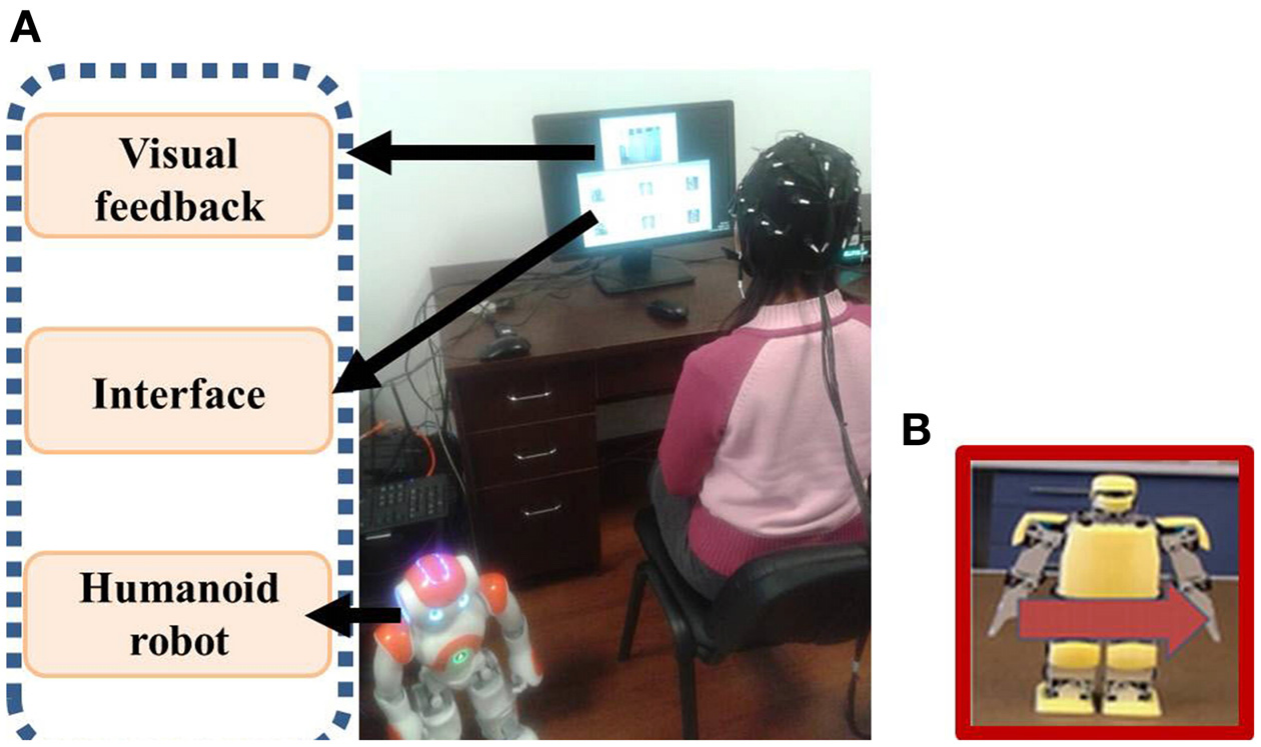
**(A)** On-line control of the NAO humanoid robot with mind in an office environment **(B)** The visual stimulus of shifting left is classified as a target.

The experiments were carried out in a normal office without electromagnetic shielding. The subjects sat in a comfortable chair and 70 cm away from a 22-inch LCD monitor displaying the N200 interface and the live video feedback, as shown in Figure 8A. The live video window was placed above the interface window with a distance to reduce mutual influences on subjects' concentrations caused by visual stimuli and live video. During the experiments, the subjects needed to stabilize their heads since the head motion may cause noises. Once a trial began, the subjects relied on live video to observe the robot status and surroundings and focused on a visual stimulus depicting robot behavior whose meaning represented their intention. When a trial was ended, the classification result was transformed to a command to activate an appropriate robot behavior. For the on-line experiments, the interval between repetitions was set 600 ms, therefore the duration of outputting a command with 3 repetitions was 5.76 s; the duration was 9.60 s with 5 repetitions. The interval between trials was set 5 s as the subjects needed this interval to have a short rest and to decide the next behavior. Figure 8B shows an example of controlling the NAO robot to shift left with mind. The supplementary material (movie clip) that records the on-line control processes of navigating a humanoid robot in an office environment with an obstacle and picking-up an object is available.

**Figure 9:**
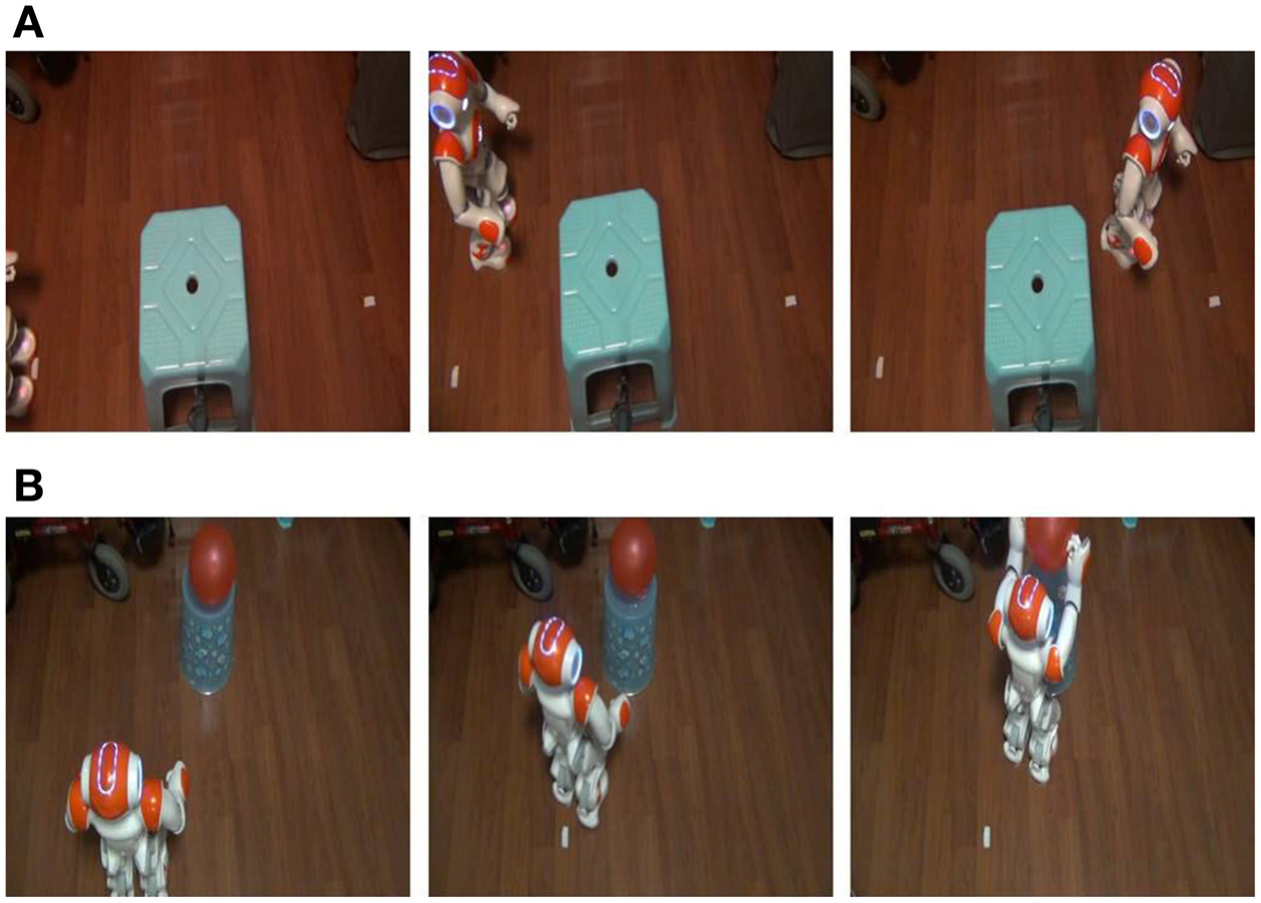
**On-line study cases of controlling the humanoid robot using the N200 model. (A)** Robot navigation with obstacle avoidance. **(B)** Robot pick-up operation.

For the navigation task, the subjects controlled the humanoid robot to walk from a start point to a destination by passing an obstacle as shown in Figure 9A. Based on the live video from the robot's camera, the subjects used N200 potentials to activate six types of robot walking behaviors defined by: walking forward for 0.2 m, walking backward for 0.15 m, shifting left for 0.15 m, shifting right for 0.15 m, turning left for 30°, and turning right for 30°. We evaluate their control performance using the following criteria: the total commands for activating robot behaviors, the total time for completing the task, the on-line control success rates, and the number of collisions with an obstacle in the three experiments. The robot may collide with the obstacle when a subject cannot appropriately estimate the distance between the robot and the obstacle from the live video or his/her intention is incorrectly detected. Table 2 lists the results herein averaged over the three experiments.

**Table 2:** **Performance of the navigation task**.

**Subject**	**Repetition number**	**Time/ command**	**Total time (s)**	**Total commands**	**On-line success rate (%)**	**Collisions**
subj1 (Y)	3	5.76	99.8	28/3	96.4	0/3
	5	9.60	144.3	30/3	100.0	0/3
subj2 (N)	3	5.76	180.8	49/3	84.5	1/3
	5	9.60	163.4	34/3	88.8	0/3
subj3 (N)	3	5.76	265.3	75/3	77.2	0/3
	5	9.60	245.6	51/3	78.4	0/3
subj4 (Y)	3	5.76	116.1	33/3	90.9	0/3
	5	9.60	154.3	32/3	100.0	0/3
subj5 (N)	3	5.76	163.4	46/3	78.2	0/3
	5	9.60	229.7	45/3	73.4	4/3

For the pick-up operation task, the subjects controlled the humanoid robot to approach a balloon and to pick up this target as shown in Figure 9B. The balloon was placed on a round table in the right front of the robot so the subjects had to move the robot to a position close enough to the table and to configure a proper orientation to pick up the balloon. For this task, the subjects used N200 potentials to activate the following defined behaviors: picking-up the target, walking forward for 0.1 m, shifting left/right for 0.1 m, and turning left/right for 15°. A task operation was classified as a failure if the pick-up behavior was activated before the robot reached the proper position. This task operation was repeated until each subject completed three successful experiments. In order to evaluate the on-line control performance, we recorded the total commands for activating robot behaviors in the three successful experiments, the average total time of completing a pick-up operation task, the average on-line success rate averaged over the three successful experiments, and the ratio of the successful experiments over the total ones.

Tables 2, 3 list the experimental results conducted by the five subjects. We compare their on-line control performance using 5 repetitions for a trial as defined in Table 1 because this repetition number documents that all the subjects achieve their accuracies over 80%. For comparing the on-line control performance, Tables 2, 3 also list the test results with 3 repetitions of a trial. We address five remarks as follows.

1. The on-line success rates differ from the off-line accuracies. The experiments of the navigation task documented that subj2, subj3, and subj5's on-line success rates (88.8, 78.4, 73.4%) were lower than their off-line accuracies (98.6, 92.8, 81.4%) when the repetition number was 5; while subj1 and subj4's success rates (100.0, 100.0%) were slightly higher than their off-line accuracies (98.6, 97.1%). The experiments of the pick-up operation task documented that subj1, subj2, and subj4's on-line success rates (95.2, 96.7, 92.3%) were slightly lower than their off-line accuracies (98.6, 98.6, 97.1%), while subj3 and subj5's success rates (100.0, 95.8%) were higher than the off-line accuracies (92.9, 81.4%).
2. Most of the subjects achieved higher on-line success rates for the pick-up operation task than those for the navigation task. The subjects subj1, subj2, subj3, and subj4 achieved the higher on-line success rates (100.0, 96.3, 95.5, 100.0%) for the pick-up operation task than those (96.4, 84.5, 77.2, 90.9%) for the navigation task when the repetition number is 3. The subjects subj2, subj3, and subj5 achieved the higher on-line success rates (96.7, 100.0, 95.8%) for the pick-up operation task than those (88.8, 78.4, 73.4%) when the repetition number is 5.
3. A lower success rate results in more total commands and longer total time for completing the operation tasks. For the navigation task, subj2 and subj3's success rates (84.5, 77.2%) with 3 repetitions were lower than the ones (88.8, 78.4%) with 5 repetitions, so their corresponding total commands for three successful experiments (49/3, 75/3) with 3 repetitions were more than ones (34/3, 51/3) with 5 repetitions and their average total times (180.8, 265.3 s) for completing each of the three successful experiments with 3 repetitions are longer than those (163.4, 245.5 s) with 5 repetitions although their duration of 5.76 s for outputting a command with 3 repetitions was shorter than the one of 9.60 s with 5 repetitions. For the pick-up operation task, when subj1, subj3, subj4, and subj5 achieved the higher success rates (100.0, 100.0, 100.0, 95.8%), they generated the fewer commands (19/3, 16/3, 20/3, 19/3). A low success rate increases the possibility of incorrectly detecting the subjects' intentions, so outputting additional commands to correct the false ones increases the number of total commands. For the three experiments, subj2 used 34 commands to navigate the NAO robot without collision with the obstacle under the success rate of 88.8%, while subj5 used 45 commands to navigate the NAO robot with 4 collisions with the obstacle under the success rate of 73.4%.
4. A repetition number of a trial plays an important role in determining a total time for completing an operation task at a high success rate. For completing three successful navigation tasks, subj1, subj4, and subj5 varied slightly their numbers of total commands (from 28 to 30, from 33 to 32, from 46 to 45) when the repetition number increased from 3 to 5. For completing three successful pick-up operation tasks, subj1, subj2, and subj3 varied slightly their numbers of total commands (from 19 to 21, from 22 to 23, from 20 to 16) when the repetition number increased from 3 to 5. For the above cases, changing the repetition number does not affect the number of total commands very much, so the duration of a trial, i.e., the repetition number determines the total time for completing an operation task. Increasing the repetition number from 3 to 5, i.e., growing the duration of a trial from 5.76 to 9.60 s, increases the total times for completing the navigation tasks discussed above from 99.8 to 144.3 s, from 116.1 to 154.3 s, and from 163.4 to 229.7 s, and the total times for completing the pick-up operation tasks from 65.4 s to 99.3 s, from 73.7 to 105.0 s, from 70.4 to 73.4 s, as listed in Tables 2, 3.
5. The experience with the designed experimental procedure is an important factor that impacts the on-line success rates. The subjects subj1 and subj4 with prior experience on the experiments achieved the high on-line average success rates of 97.9 and 95.8%, while the subjects subj2, subj3, and subj5 without prior experience delivered the relatively low success rates of 91.6, 87.9, and 78.5%.

**Table 3:** **Performance of the pick-up operation task**.

**Subject**	**Repetition number**	**Time/command**	**Total time (s)**	**Total commands**	**On-line success rate (%) for successful experiments**	**Successful / total experiments**
subj1 (Y)	3	5.76	65.4	19/3	100.0	3/4
	5	9.60	99.3	21/3	95.2	3/3
subj2 (N)	3	5.76	73.7	22/3	96.3	3/3
	5	9.60	105.0	23/3	96.7	3/3
subj3 (N)	3	5.76	70.4	20/3	95.8	3/4
	5	9.60	73.4	16/3	100.0	3/5
subj4 (Y)	3	5.76	69.1	20/3	100.0	3/5
	5	9.60	123.6	26/3	92.3	3/3
subj5 (N)	3	5.76	94.1	27/3	66.7	3/4
	5	9.60	85.0	19/3	95.8	3/5

## Discussion

### The N200 model

The work (Karakas et al., 2000b) shows that the ERP represents interplay between the oscillations that are mainly in the delta and theta frequencies. We assume that the moving bar scanning over the robot images may impact on event-related oscillations (EROs) in the temporal-parietal area, because the brainwaves acquired from this area deliver the recognizable N200 potentials which may be contributed by the theta oscillation related with orientation and attention (Karakas et al., 2000a,b). We will verify this assumption in our further research.

The interface that we design for inducing the N200 potentials has three advantages. First, the robot images as the visual stimuli are more intuitive and help the subjects understand the meanings of the stimuli better. Second, scanning the static robot images by the blue bar, instead of flashing the robot images in the traditional P300 model, allows the subjects more intensively to concentrate on a target stimulus. The subjects reported that the moving bar could cause less visual fatigue than the flashing images. Third, the proposed visual stimulus mode also induces the P300 potentials, but the amplitudes of the N200 potentials are larger and more recognizable, so we use the N200 potentials to establish the features vector to achieve better classification accuracy.

### Effects on the on-line success rates

We note that the on-line success rates vary for different operation tasks and their differences depend on the individual subjects. Here, we present the factors that affect the on-line success rates. The on-line success rates achieved in on-line control of the robot are usually lower than classification accuracies in the off-line evaluation. It would be a common fact because the off-line evaluation is an open-loop control, while the on-line task-driven control is a closed-loop control with live video feedback. Different from the off-line control, the on-line telepresence control needs a subject to coordinate his/her attention to both the live video and the visual stimuli, and especially the live video may divert a subject's attention away from the target stimulus, which decreases the on-line success rates (Gergondet et al., 2012). Especially, the poor quality of live videos may significantly deteriorate the subject's control performance. Auditory effect from the surrounding, e.g., the sound of robot's walking steps, may be another factor that distracts the subject's attention. Tidoni et al. (2014) reported that the sound of robot's walking steps delivered synchronously with the robot motion needs less time to control the robot than the asynchronous sound. In general, the success rates of the navigation task with noisy walking steps' sound are lower than those of the pick-up task. The subjects have to make a right decision based on the surrounding information sent by the live video to control the robot behavior. In addition, the subjects may get anxious when the robot falls down or collides with an obstacle caused by an incorrect command.

### Analysis of the on-line controlled tasks

We evaluate the total execution time of completing an on-line controlled task. Because the total time depends on the total commands generated by the induced N200 potentials, quickly and accurately outputting a command shortens the total time. The two factors mainly affect the total number of generated commands. The first factor is the on-line control success rate that indirectly indicates how many incorrect commands are outputted for the on-line control process. Each incorrect command causes an unexpected robot behavior that needs to be corrected by additional control commands. For the navigation task, subj2 and subj3 achieved the success rates with 3 repetitions lower than those with 5 repetitions and for the pick-up operation task subj5 achieved the success rates with 3 repetitions lower than those with 5 repetitions, so they spent more total time to complete their corresponding tasks since they outputted much more additional control commands. The second factor is the individuality of mental activities of planning in real-time to complete an operation task indicated by the following cases: 1. With 5 repetitions, subj5's brain activated fewer control commands than subj2's brain did to accomplish the pick-up operation task; 2. With 3 repetitions, subj3's brain activated fewer control commands than subj2's brain did to complete the pick-up operation task; 3. With 5 repetitions, subj5's brain activated fewer control commands than subj3's brain did to complete the navigation task.

The repetition number of a trial is a very important factor that affects the total time of an on-line task operation. The repetition number determines the duration of outputting a command. The large duration increases the total time. For example, subj1, subj4, and subj5 spent more time with 5 repetitions than with 3 repetitions to accomplish the navigation task, and subj1, subj2, and subj3 spent more time with 5 repetitions than 3 repetitions to complete the pick-up operation task, because each of them outputted the close number of total commands no matter the repetition number is 3 or 5. On the other hand, the large repetition number yields the high success rate. As discussed above, the high success rate reduces the incorrect outputs in the control process. Consequently, the few incorrect commands that need to be rectified shorten the total time. Usually, a high success rate needs the large repetition number to increase the reliability of the control system, but it increases the duration of a trial. How to determine the repetition number is an important issue because a balance between the reliability and the total time needs to be considered.

We used the robot images as the visual stimuli successfully to induce the N200 potentials with recognizable amplitudes. We implemented the proposed N200 model on the Cerebot platform to evaluate its off-line and on-line performances across five subjects. The off-line evaluations show that the average accuracy is 93.71% over the five subjects with 5 repetitions in a trial and two of the five subjects reach their accuracies of 98% with three repetitions. The five subjects participated in the navigation and pick-up operation tasks in an office environment in which the live video feedback provided surrounding information. The success rates affect the total number of commands outputted from the N200 model, the total time for completing an on-line operation task, and the number of collisions caused by the incorrect commands. Therefore achieving a high success rate has a priority using the N200 model to control the humanoid robot. As discussed above, the repetition number of a trial plays a prominent role in achieving the high success rate and shortening the duration of outputting a command. In the future work, we investigate the optimal repetition number according to a variety of on-line operation tasks.

Some research teams applied the steady state visually-evoked potential (e.g., Gergondet et al., 2012; Tidoni et al., 2014) to control a humanoid robot with live video or the motor imagery (e.g., Cohen et al., 2012) to control the robot behavior for a navigation task. In the future work, we will evaluate the performances of these models using the Cerebot platform.

### Conflict of interest statement

The authors declare that the research was conducted in the absence of any commercial or financial relationships that could be construed as a potential conflict of interest.
