# Supplementary material for: Control of humanoid robot via motion-onset visual evoked potentials
Source: Front Syst Neurosci. 2015 Jan 9;8:247. doi: 10.3389/fnsys.2014.00247 (PMC4287730; doi:10.3389/fnsys.2014.00247)
Supplement: Supplementary file 2 [file DataSheet1.ZIP › subj1_N200_offline_experiment001_figure6/Instruction of Data Sheet.pdf]

Instruction of N200 Data Sheets for "Control of humanoid robot via motion-onset visual evoked potentials"

A. xxxxx_signal.mat

This file contains a brain signal variable, NSx:

- NSx.MetaTags.SamplingFreq: sampling frequency
- NSx.Data: brain signals; each row represents a channel in the EEG cap.

B. xxxxx_NEV.mat

This file contains an event, NEV:

- NEV. MetaTags.SampleRes: sampling frequency of the I/O.
- NEV.Data.SerialDigitalIO.UnparsedData: the index of the stimulus;
  49-walking forward;
  50-walking backward;
  51-shifting left;
  52-shifting right;
  53-turning left;
  54-turning right.
- NEV.Data.SerialDigitalIO.TimeStampSec: the time point when a visual stimulus is sent.

C. The target stimuli in each trial are different and the order of targets in an experiment is: 49,50,51,52,53,54,49,50,51,52,53,54.

D. The index of the EEG cap channel

1	O2	9	TP8	17	FT8	25	FT7
2	O1	10	T8	18	TP7	26	FC3
3	OZ	11	P7	19	C3	27	F3
4	PZ	12	P3	20	FCZ	28	FP2
5	P4	13	CP3	21	FZ	29	F7
6	CP4	14	CPZ	22	F4	30	FP1
7	P8	15	CZ	23	F8	31	
8	C4	16	FC4	24	T7	32	
